# How Combined Trip Purposes Are Associated with Transport Choice for Short Distance Trips. Results from a Cross-Sectional Study in the Netherlands

**DOI:** 10.1371/journal.pone.0114797

**Published:** 2014-12-04

**Authors:** Eline Scheepers, Minke Slinger, Wanda Wendel-Vos, Jantine Schuit

**Affiliations:** 1 VU University Amsterdam, Department of Health Sciences and EMGO institute for Health and Care Research, De Boelelaan 1105, 1081 HV Amsterdam, The Netherlands; 2 National Institute for Public Health and the Environment, Centre for Nutrition, Prevention and Health Services, PO Box 1, 3720 BA Bilthoven, The Netherlands; Beihang University, China

## Abstract

**Background:**

One way to increase physical activity is to stimulate a shift from car use to walking or cycling. In single-purpose trips, purpose was found to be an important predictor of transport choice. However, as far as known, no studies have been conducted to see how trips with combined purposes affect this decision. This study was designed to provide insight into associations between combined purposes and transport choice.

**Methods:**

An online questionnaire (N = 3,663) was used to collect data concerning transport choice for four primary purposes: shopping, going to public natural spaces, sports, and commuting. Per combination of primary trip purpose and transport choice, participants were asked to give examples of secondary purposes that they combine with the primary purpose. Logistic regression analyses were used to model the odds of both cycling and walking versus car use.

**Results:**

Primary trip purposes combined with commuting, shopping, visiting private contacts or medical care were more likely to be made by car than by cycling or walking. Combinations with visiting catering facilities, trips to social infrastructure facilities, recreational outings, trips to facilities for the provision of daily requirements or private contacts during the trip were more likely to be made by walking and/or cycling than by car.

**Conclusion:**

Combined trip purposes were found to be associated with transport choice. When stimulating active transport focus should be on the combined-trip purposes which were more likely to be made by car, namely trips combined with commuting, other shopping, visiting private contacts or medical care.

## Background

Physical activity is essential for improving and maintaining health. [1] Since even small increases can lead to health benefits, promoting physical activity is a public health priority. [2], [3] One way to increase physical activity is to exchange or supplement driving a car with activity like cycling or walking. Hence, stimulating active transport by encouraging replacement of short-distance car trips with cycling or walking has become a popular policy strategy. [4] However, as far as known, the short-distance car trip has not been defined. To give policy measures a reasonable chance of success, trips that may potentially be replaced by bicycle trips should not exceed a feasible cycling distance. A distance up to 7.5 km then comes to mind since it represents a maximum of 30 minutes of cycling at an average speed; a time-span that links up with physical activity guidelines. [5], [6] In 2007, 70% of all trips in the Netherlands were shorter than 7.5 km. Of these, 36% were made by car, 34% by cycling, and 27% by walking. [7] Altogether, it seems feasible, at least for the Dutch situation, to define short-distance trips as those up to a trip length of 7.5 km.

In previous analyses, we found trip purpose to be an important predictor for use of active (cycling/walking) instead of passive (car) transport. [8] Other studies have focused on motives for transport choice in the context of single-purpose trips. For example, Carse et al. looked at characteristics associated with choosing the car versus the bicycle for commuting to work, shopping and leisure trips. [9] Commuting distance, car parking availability, access to a car and lower education levels were found to be associated with using the car for commuting. For shopping and leisure trips, access to a car and a lower education level were associated with using the car. Panter et al. studied the likelihood of incorporating walking or cycling into car journeys for commuting purposes. [10] They found an association with having to pay for parking at work, having no car parking at work and having a supportive environment for cycling and walking on the route to work. Both studies focused on explanatory variables for transport choices for trips with a single purpose. In case of more than one purpose, the trip becomes more complex [11].

One behavior, such as commuting by car, may be integrally related to another, such as bringing children to school. In our opinion, these complex trips with more than one purpose may better represent daily routine and therefore it seems evident there would be a link between combining purposes and transport choice. Few if any studies have been conducted on this association, yet insight is of interest since it will enable policy makers and developers of intervention measures to design strategies to stimulate active transport since probably other aspects should be taken into account when focusing on these combined trips than when focusing on trips with a single purpose. The current study was designed to provide insight into associations between combined trip purposes and transport choice, particularly taking into account active (cycling or walking) versus passive transport (car) and identify combined purposes that were more likely to be made by car.

## Methods

### Data collection

This study is part of the AVENUE project. An online questionnaire was used to collect data concerning transport choice (car, cycling, walking) for four primary trip purposes: shopping, going to public natural spaces, going to sport facilities and commuting. These four purposes were chosen since it was expected these purposes are of interest to policy makers and developers of intervention measures since they imply a clear set of stakeholders and partners involved in case an intervention or policy measure in this domain is considered.

Per combination of primary trip purpose and transport choice, participants were asked (4-point scale: ‘always’, ‘often’, ‘sometimes’, ‘never’) how often their transport choice for the primary trip purpose was influenced by the ability to combine trip purposes. If they answered ‘always’, ‘often’ or ‘sometimes’, they were presented with an open-ended question asking for examples of secondary purposes that they combine with a particular primary purpose (given the particular transport choice for that primary purpose).

Data collection was conducted over one calendar year, starting July 2012. Since an IRB approval is only needed when daily life of participants if influenced or participants should perform specific actions an IRB approval was not warranted and therefore not obtained.

### Study population

The study population consisted of an internet panel (N = 35,000) representative for the general Dutch population. From the panel, a random selection was made of persons at least 18 years old, since this is the minimum age to get a driver’s license in the Netherlands. Of this random selection, 8,813 participants were invited to participate in this study and received an email with a link to the questionnaire. Participants were considered eligible for the study if: 1) they were sufficiently healthy (not hampered) to use at least one of the three transport modes; 2) they made at least one short trip (max 7.5 km) a week using at least one of the transport modes; and 3) they were accustomed to travel up to 7.5 km directly from home for at least one of the four primary trip purposes. Only persons meeting all criteria received the remainder of the questionnaire. A total of 3,663 persons completed the questionnaire (response rate: 42%). Data were anonymized prior to the moment that the authors received the dataset from the owner of the internet panel and the authors did not have access to any identifying information.

### Data analyses

For every primary trip purpose, respondents were classified into one of three transport groups based on their preferred transport choice: car users, cyclists, and walkers. Their preferred choice was inferred from their frequency of using the car, cycling, or walking. When persons used two or more transport modes equally, they were categorized as a car user if one of the transport modes was a car, and as a cyclist in all other cases.

Answers to the open-ended questions concerning secondary trip purposes were coded acording to the code list from Mobility Research Netherlands. [12] Each code represented an activity on destination (e.g. shopping, sports, recreational outings). If a participant’s answer did not fit an existing code, a new code was created (Table 1). A few terms may need explanation. Shopping refers to all kind of shopping and does not distinguish between food shopping and other types. Catering facilities are those that serve food and/or drinks in public spaces. Social infrastructure refers to facilities like a library. Recreational outings include walking or cycling for its own sake or making a trip or excursion. Provision of daily requirements refers to such activities as going to a bank or post office. When a correlation of 0.50 or higher was found between two secondary trip purposes, only the secondary purpose was included in the analysis which included the largest part of the other secondary purpose. For example, walking the dog nearly always doubled with making a recreational outing, whereas a larger number of individuals that made a recreational outing were not walking the dog. In this case, only recreational outings were included in the analyses.

**Table 1:** Explanation of the used codes and overview of which codes were included in analysis.

		Primary trip purposes							
		Shopping		Public natural spaces		Sports		Commuting	
		Cycling vs. car use	Walking vs. car use	Cycling vs. car use	Walking vs. car use	Cycling vs. car use	Walking vs. car use	Cycling vs. car use	Walking vs. car use
**Secondary trip** **purposes**									
**Codes according to** **MON code list IV:** **Activity at the** **destination**	**Used codes + new** **created codes**								
To work address(e.g. factory, office, business)	Work	X	X			X			
Volunteer work									
Business trip: general(e.g. factory, office, business)	Business trips								
Business trip: one-day trip									
General participationin regular education	Education								
Education: e.g. individualcourses, driving lessons									
Education: internship									
Shopping: general	Shopping	X	X	X	X	X	X	X	X
Shopping: towncentre									
Shopping: mall									
Driving as occupation:delivery	Driving as occupation:delivery								
Driving as occupation:journeys made for	Driving as occupation:journeys made for								
Driving as occupation:other occupationaljourneys	Driving as occupation:other occupational journeys								
Taking or bringingpersons to	Taking or bringingpersons^a^								
Taking or bringingpersons at									
Leisure, general	Leisure, general								
Visiting privatecontacts	Visiting private contacts	X	X	X	X	X		X	
Visit to cateringfacility	Visit to catering facility	X	X	X	X				
Cultural activities	Cultural activities								
Use of socialinfrastructure	Use of social infrastructure	X							
Church, cemetery	Church, cemetery								
Hobby(non-athletic)	Hobby (non-athletic)								
Sports	Sports	X							
Meeting(not for business)	Meeting(not for business)								
Recreational outings	Recreational outings	X	X	X	X				
Medical care	Medical care	X							
Provision of dailyrequirements	Provision of dailyrequirements	X	X						
Government services	Government services								
Personal care	Personal care	X							
Material care(e.g. get some petrol)	Material care(e.g. get some petrol)								
	Interaction withauthorities/institutions								
	Taking or bringing items	X							
	Going to naturalenvironments	X							
	Walking the dog^b^								
	Going to garbage facility	X							
	Going to quietenvironments								
	Going to boardingpoint of public transport								
	Private contacts ondestination	X							
	Private contacts duringtrip	X	X			X			

X = included in analyses. To be included in analyses, a secondary trip purpose had to be mentioned at least 10 times (as combined with a primary trip purpose) by car users and by cyclists and/or walkers.

a Highly correlated with code 107 (private contacts during trip). When persons were coded as both 71 and 107, code 71 was deleted.

b Highly correlated with code 89 (recreational outings). When persons were coded as both 89 and 102, code 102 was deleted.

According to the literature, gender, age, educational level, household composition, neighbourhood typology and season are potential confounders for the association between trip purpose and transport choice. [13]–[16] To correct for neighbourhood typology, the dataset was merged with a dataset from ABF Research (2009). [17] Information concerning the other confounders was gathered by questionnaire or was already available from the internet panel. Base models included: gender: male/female (reference); age; educational level: low, medium, high (reference); household composition: living alone, with a partner, with children younger than 18 years, with other adults (parents, children 18 years and older, or other adults; reference); neighbourhood typology: urban-centre (reference), urban-outside centre and urban-green, village-centre and rural; and season: winter, spring, summer, autumn (reference); Regarding educational level, “low” was defined as primary school and lower general secondary education; “medium” as intermediate vocational education, higher general secondary education, and pre-university education; and “high” as higher vocational education and university (reference). The two base models were run separately for all four primary trip purposes. Per primary trip purpose, a secondary purpose was added to the base model.

### Base models



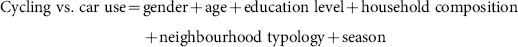


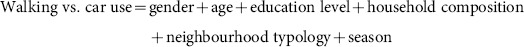



To characterize the study population, percentages of men and women, mean age, categories of educational level, household composition, neighbourhood typologies and season were calculated. Independent t-tests and Pearson’s chi-square tests were used to test for potential differences between men and women. To investigate the association between combined trip purposes and choice of transport, logistic regression analyses were used to model the odds of cycling versus car use and to model the odds of walking versus car use. For these statistical analyses, SAS 9.3 was used.

**Figure 1:**
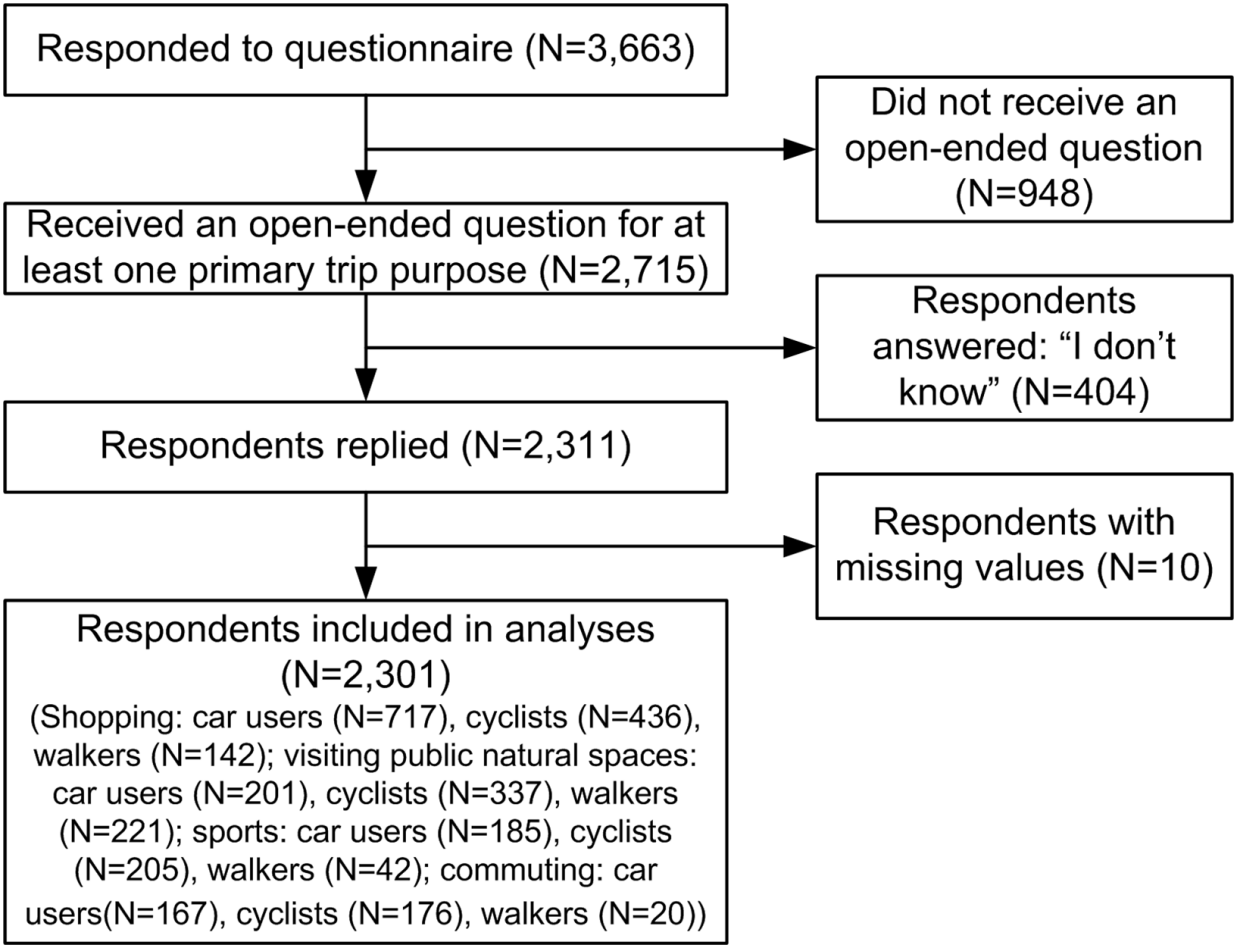
Flow-chart showing selection of study population.

To be included in statistical analyses, participants should have indicated, for at least one primary trip purpose, that their transport choice was influenced by a secondary purpose. Of the 3,663 participants who completed the questionnaire, 2715 (74%) indicated this was the case, and 2311 (63%) filled in one or more examples of secondary purposes. To be qualified as a secondary purpose, a purpose had to be mentioned at least 10 times (as combined with a primary trip purpose) by car users and by cyclists and/or walkers (Table 1). Furthermore, participants were excluded from analyses if they had missing values on one or more confounding variables. Ultimitaly, statistical analyses included data concerning 2301 respondents (Figure 1).

## Results

Table 2 shows the characteristics of the study population (N = 2301). The study population consisted of slightly more women (53%) than men (47%). Significant differences between men and women were found for age (p = 0.004), educational level (p<0.0001) and the primary trip purpose commuting (p = 0.0005).

**Table 2:** Characteristics of the study population.

	Men (N = 1088)	Women (N = 1213)
**Age (mean (SD))** *	49.7 (14.7)	48.0 (13.9)
**Educational level (%)** *		
Low	22.61	31.58
Medium	43.38	41.30
High	34.01	27.12
**Household composition (%)**		
Alone	20.04	21.19
Partner	42.19	39.90
Children <18 years	20.95	24.48
Other adults (children ≥18 years, parents, or other adults)	16.82	14.43
**Neighbourhood typology (%)**		
Rural & village centre	39.61	40.56
Urban-green & urban-outside centre	51.29	52.84
Urban-centre	9.10	6.60
**Season (%)**		
Winter	26.56	26.30
Spring	20.77	22.09
Summer	25.74	25.97
Autumn	26.93	25.64
**Primary trip purpose: Shopping**	93.66	93.32
**Primary trip purpose: Public natural spaces**	29.41	36.27
**Primary trip purpose: Sport**	76.65	75.60
**Primary trip purpose: Commuting** *	47.89	45.18

Educational level: low (primary school and lower general secondary education), medium (intermediate vocational education, higher general secondary education and pre-university education) and high (higher vocational education and university). Neighbourhood typology: rural (low density or few facilities), village-centre (higher density or more facilities than rural neighbourhoods), urban-green (predominantly residential areas, with density lower than the mean density based on housing supply, or new housing estate neighbourhoods), urban-outside centre (larger distance from the city centre, but with higher density than the ‘urban-green’ neighbourhoords, density of these neighbourhoods is most of the time also higher than in the urban-centre neighbourhoods), and urban-centre (city centres as well as some neighbourhoods just outside the centre).

*Significant difference between men and women (<0.05).

Table 3 shows the odds ratios (OR) and their 95% confidence intervals (CI) for the secondary trip purposes associated with transport choice per primary purpose. The remainder of this section will address results separately for the four primary purposes.

**Table 3:** Association between secondary trip purposes and transport choice per primary trip purpose (OR (95%)).

	Primary trip purposes							
	Shopping		Public natural spaces		Sports		Commuting	
	Cycling vs. Car	Walking vs. Car	Cycling vs. car	Walking vs. Car	Cycling vs. car	Walking vs. Car	Cycling vs. car	Walking vs. Car
**Secondary trip purposes**	**OR (95% CI)**	**OR (95% CI)**	**OR (95% CI)**	**OR (95% CI)**	**OR (95% CI)**	**OR (95% CI)**	**OR (95% CI)**	**OR (95% CI)**
**Work**	**0.61 (0.43–0.85)**	**0.28 (0.14–0.54)**			**0.22 (0.10–0.49)**			
**Shopping**	**0.57 (0.41–0.79)**	**0.43 (0.23–0.78)**	0.83 (0.57–1.21)	**0.38 (0.25–0.58)**	0.84 (0.55–1.30)	**0.36 (0.18–0.75)**	1.65 (0.92–2.94)	0.75 (0.21–2.69)
**Visiting private contacts**	0.94 (0.74–1.21)	**0.36 (0.23–0.55)**	0.75 (0.51–1.09)	**0.29 (0.18–0.46)**	1.13 (0.70–1.83)		0.56 (0.31–1.01)	
**Visiting catering** **facilities**	1.85 (0.97–3.52)	**2.79 (1.21–6.41)**	1.24 (0.69–2.25)	0.73 (0.36–1.48)				
**Social infrastructure** **facilities**	**3.54 (1.97–6.35)**							
**Sports**	1.10 (0.69–1.73)							
**Recreational outings**	**3.60 (2.26–5.72)**	**10.62 (6.08–18.54)**	0.73 (0.47–1.14)	**1.72 (1.11–2.68)**				
**Medical care**	**0.40 (0.22–0.71)**							
**Provision of daily requirements**	**1.74 (1.05–2.89)**	**2.12 (1.09–4.15)**						
**Personal care**	1.40 (0.58–3.39)							
**Taking or bringing** **items**	1.01 (0.51–2.03)							
**Going to natural** **environments**	1.55 (0.67–3.60)							
**Dropping garbage**	0.68 (0.33–1.39)							
**Private contacts on** **destinations**	1.09 (0.48–2.44)							
**Private contacts during** **trip**	1.07 (0.70–1.64)	**2.62 (1.45–4.74)**			0.44 (0.21–0.91)			

Adjusted for gender, age, education level, household composition, neighbourhood typology and season.

Abbrevations; OR = Odds Ratio indicating the odds to use active transport modes for a short trip; 95%CI = 95% confidence interval; significance was tested at α = 0.05.

Models were only run when a secondary purpose was mentioned at least 10 times (as combined with a primary trip purpose) by car users and by cyclists and/or walkers.

### Shopping

Persons who combined shopping with commuting (OR = 0.61, 95%CI: 0.43–0.85), other shopping (OR = 0.57 95%CI: 0.41–0.79), or seeking medical care (OR = 0.40, 95%CI: 0.22–0.71) were more likely to use the car than bicycle. On the other hand, persons combining shopping with trips to social infrastructure facilities, recreational outings, or trips to facilities for provision of daily requirements were more likely to cycle than using the car. For walking vs. car use, it was found that persons combining shopping with commuting (OR = 0.28, 95%CI: 0.14–0.54), other shopping (OR = 0.43, 95%CI: 0.23–0.78) or visiting private contacts (OR = 0.36, 95%CI: 0.23–0.55) were more likely to use the car than go walking. Persons were more likely to walk than using the car when combining shopping with going to catering facilities, making recreational outings, going to facilities for provision of daily requirements or meeting with private contacts during a trip.

### Public natural spaces

No significant associations were found for cycling vs. car use in trips to public natural spaces. Modeling walking vs. car use showed that persons were more likely to use the car when combining this primary trip purpose with shopping (OR = 0.38, 95%CI: 0.25–0.58) or visiting private contacts (OR = 0.29, 95%CI: 0.18–0.46). However, persons combining this primary trip purpose with a recreational outing were more likely to walk.

### Sports

Persons combining sport trips with commuting (OR = 0.22, 95%CI: 0.10–0.49) were more likely to use the car than go cycling. When combining sport trips with shopping (OR = 0.36, 95%CI = 0.18–0.75), persons were more likely to use the car than walk.

### Commuting

No significant associations between secondary trip purposes and transport choice were found.

## Discussion

Transport choice was found to significantly associated with several combinations of primary and secondary trip purposes. Main results were that primary trip purposes combined with commuting, shopping, visiting private contacts or medical care were more likely to be made by car than by cycling or walking. Combinations with visiting catering facilities, trips to social infrastructure facilities, recreational outings, trips to facilities for the provision of daily requirements or private contacts during the trip were more likely to be made by walking and/or cycling than by car.

### Passive transport: car use

Car use for primary trips combined with commuting or shopping could be explained by the possibility of carrying heavy goods. [18] Additionally, a person’s type of employment could contribute to this choice of the car, since persons wearing a suit are less likely to cycle. [19] Another explanation for choosing the car could be distance to the next destination. In our previous study, where we looked at short trips made for shopping, commuting, taking or bringing persons or sports, we found that short car trips had a longer average distance (3.3 km) than short walking trips (0.8 km). [8] Due to these shorter trips, it becomes less likely a secondary purpose is on the same route as the primary destination for walking trips. Besides, additionally walking is more strenuous than using the car. Other factors that could explain choosing the car are speed, flexibility, personal space, and safety. [9] For the combination of sport trips with commuting, time constraints could also influence transport choice. Persons going to sport facilities before or after work might not have enough time to use the bicycle. Additionally, type of sport and its necessary clothing and equipment could lead to taking the car over the bicycle. Gyms (e.g. fitness clubs) are often located in town or village centres whereas sports clubs (e.g. hockey, tennis) often are located on the edge or outside an urban or residential neighbourhood. Since urbanization and displacement of sports facilities to the edges of cities may well impede physical activity, active transport could be encouraged by, for example, creating sports and exercise facilities within neighbourhoods [20], [21].

### Active transport: cycling and walking

Persons combining primary trips with recreational outings were more likely to cycle or walk than using the car. Enjoying fresh air or health are reasons for choosing active transport over the car. [22] Shopping less heavy products when combining shopping with recreational outings could also explain this active transport use. The higher active transport use, when combining visiting public natural spaces with recreational outings could be explained by the possibility to walk the dog. Since a high correlation was found between walking the dog and recreational outings only recreational outings were included in the analyses. However, 72% of the persons who combined walking trips to public natural spaces with recreational outings also walked the dog. This in contrary to 50% making this combined trip by car.

Combinations of shopping with trips to social infrastructure facilities or facilities for provision of daily requirements were also more likely to be made by active transport. An explanation could be that these secondary destinations are often within walking distance from shopping facilities. Millward et al. showed that in Canada the most common walking trips to commercial destinations were trips to restaurants and bars, grocery stores, shopping centres and banks. [23] These destinations were within walking distance, since most walks were shorter than 600 m, and very few exceed 1200 m. Another explanation could be that these trips included small errands such as withdrawing money or posting a letter (i.e. provision of daily requirements).

### Strengths and limitations

A questionnaire specially designed to investigate transport choice was used, enabling us to study transport choice and combined trip purposes in more detail than when using secondary data sources like Mobility Research Netherlands. In addition, data was collected for all days of a full year, enabling correction for seasonal influences.

Participants were asked about their addition of secondary purposes to primary purposes However, forty to sixty percent of the participants that gave some examples of these secondary purposes, answered that they only sometimes combine this secondary purpose with the primary purpose. In addition, the order in which these combined trips were made was not determined, nor was the distance of the secondary trips. Future research should focus on the distance and order of these combined trips as well as the frequency of combining these trips, since this information is needed to develop suitable policy measures.

The Mobility Research Netherlands coding list was used to code the secondary trip purposes and thereby contributed to the quality of the study. However, a limitation could be that multiple activities on destination were merged into several categories. For example “shopping” included shopping for groceries as well as clothes. Further research should separately examine these different kinds of shopping trips.

In the present study, the sample size was too small to allow stratification by, for example, gender or age groups. Additionally, since this study was a first exploration of the association between combined trip purposes and transport choice, the focus was only on the most important confounders. Other factors such as physical activity, health, satisfaction with the living environment, or owning a car could also affect this association. Future research should investigate the influence of other confounders as well as using larger sample size allowing stratification for potential interaction effects. In this study 2154 persons made short trips for shopping, which resulted in more answers on the open question concerning secondary purposes than for the other primary purposes. These larger numbers enabled us to analyze the association between combined trip purposes and transport choice for more combinations of trip purposes. Future research should also focus on these specific secondary purposes in combination with the other primary trip purposes.

Several secondary trip purposes were excluded for analysis because they were mentioned fewer than 10 times by the study population. For example, business trips were relevant for people using the car to commute. However, for walking and cycling this secondary trip purpose was not applicable and thus transport choice could be difficult to change. However, more information about these trip purposes is needed in order to develop suitable interventions and measures for stimulating active transport modes.

Finally, valuable information concerning transport modes used for several combined-trip purposes was gathered. However, no information was available on people’s motives for transport choices or their motives for making particular combinations of trip purposes. Future research is needed to elucidate these motivations.

### Conclusion

Aim was to provide insight into associations between combined trip purposes and choice of transport choice. The results suggest that combined trip purposes should be taken into account when stimulating a shift from car use to cycling or walking. When stimulating active transport, focus should be on the combined trip purposes that were more likely to be made by car, namely trips combined with commuting, other shopping, visiting private contacts or medical care. However, more detailed information (e.g. motivations, trip characteristics) about these combined trip purposes is needed to allow development of suitable interventions or policy measures.
